# PReS-FINAL-2334: Chronic recurrent multifocal osteomyelitis and tnf-αlfa inhibitors

**DOI:** 10.1186/1546-0096-11-S2-P324

**Published:** 2013-12-05

**Authors:** M Alberdi-Saugstrup, SM Nielsen

**Affiliations:** 1 department of pediatric rheumatology, Rigshospitalet, Copenhagen, Denmark

## Introduction

Chronic recurrent multifocal osteomyelitis (CRMO) is an inflammatory disorder of unknown etiology, characterized by nonbacterial inflammatory bone lesions. The bone lesions can be unifocal or multifocal, and have an either uniphasic or recurrent course.

Non-steroidal anti-inflammatory drugs (NSAIDs) and Glucocorticoid have been reported to be effective in some cases, and more recently tumor necrosis factor-alpha (TNF-α) inhibitors have shown effect in more treatment resistant cases.

## Objectives

To describe a cohort of CRMO patients and characterize treatment effect.

## Methods

Data from all patients diagnosed with CRMO from 2002 to 2011, at the east Danish specialized pediatric rheumatology unit, were collected through review of medical records.

The effect of treatment were evaluated by the clinical symptoms, blood analysis and by Magnetic Resonance Imagining (MRI).

## Results

From January 2002 to December 2011, twenty-five children under the age of 16 years were diagnosed with CRMO. 16 females, 9 males. The most frequent foci were in the tibia (8), femur (7), clavicle (7) and fibula (4). 56% of the patients had unifocal affection and 24% could be diagnosed with SAPHO syndrome.

Seven patients (28%) had sufficient effect of treatment with NSAIDs.

Only three out of 18 patients treated with Methrotrexate (MTX) had sufficient effect of the drug.

15 patients were treated with TNF-α inhibitors. 12 patients changed to a 2.line and three to a 3. line TNF-α inhibitor, due to either insufficient effect or adverse events.

The overall effect of TNF-α inhibitor were very good. The effects of treatments are shown in Table 1.

**Table 1:** 

Table 1	ptt.	%
Sufficient clinical effect of MTX (n:18)	3	17 %

Sufficient clinical effect of TNF-α (n:31)	18	58 %

Regression on MRI, MTX (n:9)	4	44 %

Regression on MRI, TNF-α (n:13)	11	84 %

## Conclusion

The gender and age distribution of our patients resample that of the literature. In contrast we found a limited amount of foci, 1,6/patient. A large number of our patients developed SAPHO-syndrome. This could be caused by selection bias, since we are a highly specialized unit, with primarily more complicated patients being referred.

MTX had a disappointing effect on our cohort, in contrast to that of TNF-α inhibitors.

A treatment-resistant group of patients showed good effect from TNF-α inhibitors, both clinical and on MRI.

In conclusion TNF-α inhibitors seem to be a relevant treatment, with good possibility of effect, both clinical and radiographic.

## Disclosure of interest

None declared.

